# Unspoken expectations and situational participation: a qualitative study exploring the instantiation of next of kin involvement within the trust model

**DOI:** 10.1186/s12913-024-11338-9

**Published:** 2024-07-30

**Authors:** Ruth-Ellen Slåtsveen, Torunn Wibe, Liv Halvorsrud, Anne Lund

**Affiliations:** 1 Department of Rehabilitation Science and Health Technology, Faculty of Health Sciences, OsloMet- Oslo Metropolitan University, St. Olavs Plass, PO Box 4, Oslo, 0130 Norway; 2 Centre for Development of Institutional and Home Care Services in Oslo, PO box 4716, Oslo, N-0506 Norway; 3 Department of Nursing and Health Promotion, Faculty of Health Sciences, OsloMet- Oslo Metropolitan University, St. Olavs Plass, PO Box 4, Oslo, 0130 Norway; 4 Department of Rehabilitation Science and Health Technology- Occupational Therapy, Faculty of Health Sciences, OsloMet- Oslo Metropolitan University, St. Olavs Plass, PO Box 4, Oslo, 0130 Norway

**Keywords:** Collaboration, Frontline workers, Home-based healthcare, Interdisciplinary team, Next of kin, Primary healthcare, Service organisation, Trust model

## Abstract

**Background:**

Demographic changes, such as an increase in older adults, present a challenge to the healthcare service’s current capacity. Moreover, the need for healthcare personnel is rising, while the availability of labour is dwindling, leading to a potential workforce shortage. To address some of these challenges, enhanced collaboration between home-based healthcare frontline workers, service users, and next of kin is a necessity. The trust model is an organisational model where home-based healthcare services are organised into smaller interdisciplinary teams aiming to tailor the services in collaboration with service-users and their next of kin’. This study explores how the next of kin and frontline workers perceive and perform involvement in making decisions regarding tailoring the services for the users of home-based healthcare services organised after the trust model.

**Methods:**

Four in-depth interviews and 32 observations were conducted, and thematic analysis was employed to identify meaningful patterns across the datasets.

**Results:**

The results are presented as two themes: (i) unspoken expectations and (ii) situational participation. The results highlight the complex nature of next-of-kin involvement and shared decision making, raising questions about meeting expectations, evaluating available resources, and developing sustainable involvement processes.

**Conclusion:**

This study indicates that despite of an interdisciplinary organisational model aiming for shared decision making as the trust model, the involvement of next of kin continues to be a challenge for frontline workers in home-based healthcare services. It also points to the importance of transparent communication and how it is deemed essential for clarifying implicit expectations.

**Supplementary Information:**

The online version contains supplementary material available at 10.1186/s12913-024-11338-9.

## Background

Home-based healthcare is an essential part of primary care which in turn enables health systems to support a person’s health needs – from health promotion to disease prevention, treatment, rehabilitation, palliative care, and more [1]. It aims to deliver healthcare in a way that is centred on people’s needs and respects their preferences. Achieving access to quality essential healthcare services to ensure healthy lives and promote well-being for all at all ages is one of the United Nation’s sustainable Developments Goals [2]. Demographic changes, including a higher percentage of older adults, challenge the current capacity of healthcare services [3, 4]. The need for frontline workers is increasing, while access to labour is slowing down and is eventually expected to stop [4]. Hence, the demographic changes not only centre around the increasing number of older adults but also a reduction in the workforce [3, 5]. In this study, “frontline workers” is used as a collective term for those delivering the home-based healthcare service. This includes case managers, occupational therapists, physiotherapists, nurses, and other healthcare staff.

In Norway, home-based healthcare forms part of the universal welfare model, and the service is offered to and used by all groups of citizens [6]. Home-based healthcare is provided by paid frontline workers in the homes of persons in need of healthcare, covering a range of activities, from short-term rehabilitation to long-term assistance with basic daily activities and advanced treatment of chronic or terminal illnesses [7]. Home-based healthcare is a multidisciplinary service consisting of different frontline workers organised into different sections within the municipalities, and it is a complex interactive process involving the service user and the next of kin [7]. Most Norwegian municipalities use a purchaser-provider model of home-based healthcare services, inspired by new public management. Notably, the purchaser-provider model distinguishes between those responsible for the allocation of services and the frontline workers who deliver them [8, 9]. In this structure, the organisation of home-based healthcare is divided into different sections, with one section responsible for service allocation, another comprised of nurses and healthcare staff, and a third section with occupational therapists and physiotherapists. Thus, the services involve reporting and control routines, which are purported to divert much of the focus away from the service delivery, potentially disadvantaging the service users [8–11].

Norwegian national policies outline a need for new solutions, and reconstructions in the healthcare services focusing on better planning, strengthened prevention, and more targeted services [4, 5]. Furthermore, in line with the United Nations’ Sustainable Developments Goals, the focus is to ensure that everyone who needs it has access to good and safe services, which is achieved through better use of the collected resources [2, 5, 12]. This includes collaboration between the frontline workers and the service users and their next of kin. The next of kin’s care contribution corresponds to around 50% of the healthcare services in Norway [13, 14]. Norwegian national health policies promote an expectation of a larger degree of involvement of next of kin in healthcare services [4, 15].

Some municipalities in Norway have implemented an organisational model in home-based healthcare services called the *trust model*, and by that also altered the purchaser-provider model. The trust model outlines a reorganisation into smaller self-managed interdisciplinary teams, with the case manager as an equal member, making decisions in continuous collaboration with the other team members, the service users, and their next of kin [16–18]. The purpose of this reorganisation is to enable professional judgments and rapid responses to the changing needs of service users, rather than being hindered by time-consuming bureaucratic procedures [8, 19]. In this way, the frontline workers are trusted to use their expertise and experiential knowledge, thereby enhancing the flexibility and individual tailoring of services. Service users and their next of kin are also expected to receive closer follow-up from a reduced number of frontline workers, hence enhancing service user knowledge, the feeling of being heard and understood, improve collaboration and shared decision making [20]. The involvement of the service user and their next of kin is described as an important tool to ensure equal, comprehensive, and coordinated services. Involvement is here understood as a way of coproducing the services [17, 18, 20]. The goal is for the next of kin to experience well-functioning and predictable cooperation with the services, which is also regulated by law [18, 21–23].

Next of kin involvement is described to be required in the design and implementation of healthcare when there is a need and a legal basis for it [17, 18, 21, 23, 24]. Thus, the next of kin should be involved as providers of in-depth knowledge and insight regarding the service user, valuable information that is otherwise difficult to gather [17]. The frontline workers are encouraged to show the ability and willingness to meet and cooperate with next of kin on an equal basis, where their experiential knowledge is acknowledged and valued in dialogue and cooperation [17, 18, 21].

A needs-led research process involving service-users, next of kin, leaders, and frontline workers showed a need for in-depth knowledge related to the trust model’s intention regarding the involvement of next of kin [25]. Furthermore, the literature calls for exploring how the trust model contributes to more flexible and individual tailored services through the more thorough involvement of service users and next of kin [16, 20, 26]. Moreover, there is limited knowledge about how frontline workers address the needs of both service users and next of kin when allocating services or in collaborating, involving, or using next of kin’s expertise to deliver flexible and individually tailored services [24, 27].

To advance existing knowledge, this study explores how the next of kin and the frontline workers perceive and perform involvement in decision making regarding tailoring the services for the service users of home-based healthcare services organised after the trust model. This study aims to address the research gap concerning how frontline workers address the needs of both service users and next of kin in service allocation, collaborating, involving, or using next of kin’s expertise to deliver flexible and individually tailored services, as intended in the trust model. Additionally, it seeks to understand the experiences of involvement and collaboration with next of kin within interdisciplinary organisational models, like the trust model in home-based healthcare.

## Methods

### Study setting

The study used a qualitative design based on in-depth interviews and observations conducted in a larger Norwegian municipality [28]. The interview guide used was developed for this study (see supplementary file).

### Data collection

Between August 2022 and March 2023, four in-depth interviews were conducted with next of kin of service-users of the home-based healthcare services. Due to Covid-19, these interviews were conducted over the phone following the participants’ wishes, making the participation more feasible for them. The next of kin were recruited through frontline workers who distributed both verbal and written information about the study along with the first author’s contact information. There were no specific inclusion criteria other than being next of kin to a person receiving home-based healthcare in that municipality. By coincidence, it ended up being a demographical homogenous group of next of kin. All four were retired, children or children-in-law of the service users, and not living with them. All of them cared for persons with dementia, both in the early and advanced stages of the disease.

To achieve a broader and wider data material to analyse a choice was made to combine these interviews with data material from 32 observations that were already conducted. These were observations of different interdisciplinary meetings within the home-based healthcare services, and meetings between the leaders of home-based healthcare, in which service user cases were discussed and next of kin were mentioned. These observations were conducted between March 2021 and April 2022, involving interdisciplinary participation from home-based healthcare services as leaders from different levels, nurses, occupational therapists, physiotherapists, case managers, and other healthcare staff. Observations were conducted digitally due to Covid-19 restrictions prohibiting in-person contact outside one’s own family. No observation protocol or guideline was used. The focus was on how frontline workers discussed and collaborated regarding the service users. The digital solution enabled almost verbatim transcription of the conversations during the meetings, rendering the data material quite similar to the material from the interviews with the next of kin.

## Data analysis

The first author conducted and transcribed all the interviews and was present during every observation. The three other authors were alternately present in the observations, while all four authors made written observation notes during the meetings. For validation, the observation notes were shared and discussed within the author group. The observation was performed during the COVID-19 pandemic and was therefore done digitally by participating in the service’s digital meetings. This made it possible to take verbatim notes from the conversations.

Thematic analysis was applied to endorse the process of researcher subjectivity and the situated generation of knowledge to report patterns of meaning across the data from the interviews and observations [29]. The analysis was conducted by employing an inductive approach, without relating data to existing theory. However, exploring how the next of kin and the frontline workers perceive and perform involvement in decision-making regarding tailoring the services for the service users of home-based healthcare services organised after the trust model was prominent when analysing. All four authors individually went through the four interviews with the next of kin to become familiar with the material and perform preliminary coding. Comments were written in the material by all the authors, and the authors discussed the material before the first author continued the analyses by selecting statements, coding, and grouping the codes from the interviews. The first author then reviewed all 32 observations and analysed them using an approach based on the preliminary analyses of the four interviews. Statements by the leaders and frontline workers related to next of kin were extracted from the observations, coded, and grouped. The four authors then met to review all statements, codes, and groupings from the interviews and the observations together and form preliminary theme names [29]. Every meeting and discussion led to a deeper and more nuanced understanding of the identified patterns. Table 1 provides a few examples of extracted statements, codes, and the themes.

**Table 1:** Mapped extractions of statements, codes, and themes

“No, it’s never the other way around. Never! We’ve talked a lot about how strange it is that we never get any feedback, and questions about how they see it, how they observe him/her”. (interview next of kin)	A need for information	Unspoken expectations
“And we would like to do what we are advised, but it would have been nice to have a little more conversation about it then, if there is something relevant”. (interview next of kin)		
“No, in other words, simply when they assess things, they can contact us”. (interview next of kin)		
“The consequence of a person being demented and reluctant to accept services… How do we handle that in practice, what does the district do and what do we as next of kins do then? How can we cooperate?” (interview next of kin)	A wish for more collaboration	
“Such type of collaboration has been difficult to achieve. But, I do understand, it’s a lot… they are constantly running…. so there’s a dilemma between understanding them and having a need oneself.” (interview next of kin)		
“The start was completely overwhelming, I had never dreamed of getting such a good offer. You hear about all the horrible things, but I must say that they deserve honour in relation to what happened then. And they contacted me”. (interview next of kin)	Shared responsibility	Situational Participation
“The things we had negative responses to, we brought up and it was corrected. We had a very nice dialogue”. (interview next of kin)		
Next of kin needs to assist with the purchase of fire-retardant clothing. There is a critical situation related to fire safety, and it seems that there has not yet been a dialogue with the next of kin about the situation, but the service is considering contacting them. (from observation)	Task related involvement	
“Well, at least we could be asked if we want to be contacted. I don’t know if that might feel too much or burdensome for others… I would rather be contacted and coordinated than for us to operate on the outside and spend a lot of time and effort figuring out things that could easily have been clarified” (interview next of kin)	Shared decision making	
“We need to find out if we can work as a team with the next of kin”. (from observation)	Playing at the same team	

The author group consists of occupational therapists and nurses with different clinical backgrounds. Furthermore, together they possess extensive experience in research. The first author has 19 years of experience in home-based healthcare services and is therefore well acquainted with the research field.

## Results

The analysis revealed that involvement was an important element in creating flexible and individually tailored services. The next of kin and the frontline workers demonstrated a common interest and wanted to do what they perceived to be in the best interest of the users. The results showed the complexity of the involvement of next of kin, both in terms of how it was perceived and how it was carried out, with themes emerging: (i) unspoken expectations, and (ii) situational participation. The first theme has evolved from analysis based on the next of kin interviews and the second theme stems mostly from the observational data.

### Unspoken expectations

Involvement seemed to be an active and conscious action done by both next of kin and frontline workers. The next of kin narrated situations in which they initiated the involvement intending to create a better everyday life for the service users. However, the results also pointed to an unspoken expectation that frontline workers should initiate these conversations and collaborations, which the next of kin viewed as necessary. Consequently, when these expectations went unfulfilled, the need to be involved became a principle and, to some extent, a struggle for the next of kin. One next of kin exemplified this by saying:

*We almost had to force ourselves to a meeting*,* and it was a meeting that was more about what it was going to be like when he got home. We had been waiting for them to contact us*,* telling us what the arrangement would be like when he got home.*

The need to receive information about what was happening or should be expected next regarding the service users appeared to be a fundamental factor for the next of kin, and the results showed that they had a desire and an expectation of more extended and more frequent information from the services than they received. The need for information also seemed to be a means of creating a sense of security for themselves to confirm that the services were tailored to the service user’s needs and that the service user was thus taken care of in an adequate manner.

This is also reflected in the act of control performed by the next of kin. In the interviews, various narratives highlighted how this control was carried out and how some of them experienced losing that control and thus felt insecure on behalf of the service user. The following excerpt concerns a next of kin who was responsible for dosing and administering medications to the service user but who then had to relinquish the responsibility to the frontline workers:

*I had complete control over that. So*,* I let them [the frontline workers] know that I’m kind of worried about handing over the responsibility*,* but I was sort of told that I didn’t have to be worried. Then I say okay*,* I’m going to do random controls. Then*,* I discovered last year that they had missed giving the medication. It was forgotten*,* and I reacted strongly…*.

Several next of kin talked about involvement as a means of gaining information and insight into the various services available in the municipality for the service users. This was important information that could help them feel more confident about the choices they often have to make on behalf of the service users. Many pointed out that it was just coincidences that led to their receiving of information about various services.

These results underscore the expectations of the next of kin that frontline workers would give them sufficient information and help them understand the information. This seemed to be related to the need to know what the best decisions were, which may foster confidence in the accuracy of the chosen options. One next of kin said:

*We also need to get insight into their assessment*,* if we are the ones who have to apply for services then we need to get some more information*,* because we don’t know the different services any well- It’s completely unploughed field for me really… And we want to do what we’re advised to do somehow…*.

It appeared that a shortage of information created uncertainty and insecurity among next of kin and that the expectation and involvement regarding access to information was about gaining a better overview and thus avoiding the experience of insecurity.

Another aspect closely related to involvement from the next of kin was the need to require services or to set certain guidelines. The starting point of the next of kin seemed to be one in which they were unsatisfied with their experiences. One next of kin described a situation in which the service user was not receiving help with personal hygiene for an extensive period, ultimately affecting the service-user’s health condition. The next of kin perceived that they were expected to have asked for the intervention in question, rather than an assessment that should have been made by the frontline worker.

Unspoken expectations between the next of kin and the frontline workers seemed to drive the next of kin’s desire to involve the services. Many next of kin complemented this understanding by suggesting that they took on the responsibility of involving the service because they felt compelled to assess the service user’s needs. It appeared that a common denominator for the interviewed next of kin was a desire for involvement to proceed in a different way than experienced ones—being able to collaborate on tailoring the services, not just being contacted to exchange information. One next of kin said:

*I missed that they talked to us*,* not just change the service without us knowing … The consequence of a person being demented and refusing to accept services; How do we deal with it in practice? What* do *the services do*,* and what could we do as next of kin? How can we work together? That is the conversation that I think we might need from time to time. It’s a little bit more ‘yes*,* but what now?”*

Although the next of kin experienced their unspoken expectation of being involved unmet, they seemed to perceive that the frontline workers were pressed for time and lacked personnel. The next of kin acknowledged that the frontline workers’ challenging work situation could impact service delivery and involvement. They conveyed their willingness to adapt as required and expressed openness to collaboration. Furthermore, they expressed a desire to contribute to improving the service provided to the service user, with the ultimate goal of ensuring optimal care and experience. One next of kin shared an occasion in which cooperation and more flexibility might have had a positive outcome for the service user:

*We asked if she could participate in daycare for people suffering from dementia*,* and then … When she finally agreed … there is a requirement that she must apply for it! She won’t accept any services! How can … And then*,* I asked if we could be allowed to get a trial visit*,* instead of having to wait half a year for an application approval*,* right? … to be able to do a visit when she was in a good mood*,* just to use the opportunity to get her there. That could have been an opportunity; it creates possibilities instead of limitations.*

This wish for more collaboration emphasised more potential for tailored services than the experience of working separately in parallel with each other, which made the next of kin feel on the outside. Hence, much time and effort were spent on things that frontline workers and next of kin could quickly solve together. Situations in which the next of kin experienced being involved more collaboratively were highlighted as positive:

*Yesterday*,* we agreed on how to communicate with her … We’re not using the word long-term stay*,* we say ‘you’ve been given the opportunity to be in a place where you can feel well*,* and getting further training so that you recover’*,* and the personnel say the same things as we say. Then*,* we have that dialogue we want*,* right. And we’ve missed that*,* this collaboration …*.

### Situational participation

The frontline workers talked about involving the next of kin, and their objectives for involvement corresponded to those of the next of kin. One of the most mentioned objectives was the need for more information about the service user’s situation and insight into how next of kin experience the situation. Involvement through information gathering was emphasised as important in creating tailored services, being flexible, and adapting the services to the service user’s needs. The next of kin were also involved in their assessment visits. However, for the next of kin, the crux of the matter is obtaining information about what is important so that the services are tailored to their needs and wishes.

Furthermore, the results showed that there was not always correspondence between the information the frontline workers collected from next of kin and what they believed to be correct. The dilemma that occurred between the different perceptions of the situation and the service users’ needs also seemed to arise from unmet and sometimes unspoken expectations and different views on which services and care were necessary, as illustrated in the following excerpt:

*It has been difficult to offer the help the daughters expected*,* and the same is happening at the nursing home. I have talked with them*,* and I understand the concern*,* but they do not want to send the service user home with the help we can offer and have until now*,* nor do they want a long-term place. They come up with many arguments as to why. What they tell us is that the service user needs predictability and to feel safe; it also stems from the fact that the daughters have the same needs.*

The frontline workers described having the next of kin “playing on the same team” as they did, which can be understood as expecting the next of kin to take responsibility for information or specific tasks that the services needed. The next of kin were needed for their knowledge of the service user as a person and to carry out practical tasks based on the needs of the service. In a meeting, it was said;

*I have had a dialogue with the daughter. I have not been able to visit the user at home because of covid. However*,* I did the assessment over the phone with the daughter.*

The findings show that it involved different information and related to different situations, such as next of kin assisting with mapping the service user’s needs, serving as intermediaries between the services, and serving as language interpreters between the service users and the service if there were language challenges. This was also true even when the next of kin indicated that they did not wish to serve as liaisons. In one meeting, a part of a conversation about this went like this:

Frontline worker A: *He lives in a very small apartment*,* so there may be some challenges there. He doesn’t speak Norwegian*,* don’t think he speaks English either*,* so his daughter will attend the visit … He may need follow-up during the first period of being home …*.

Frontline worker B: *Bills and mail can be difficult when he gets home. It says in the journal that the daughter does not want to be a mediator …*.

Another example highlighted was the service’s need to obtain information from other services, such as hospitals and GPs. The next of kin felt required to bring this information between the services. A next of kin said:

*We must be the ones to bring valuable information between the services. They constantly ask us about things*,* and something has to be wrong somewhere when they are depending on us for this information.*

## Discussion

The intention of the trust model encourages collaboration with next of kin to tailor home-based healthcare services when service users are unable to do so themselves [18, 20]. Political guidelines support this approach to reduce the burden on next of kin and improve the quality of life for service users by meeting a well-functioning and predictable collaboration with the services [16, 19]. This study explores how next of kin and frontline workers perceive involvement in decision making regarding tailoring services for service users, and how this is performed within interdisciplinary teams organised according to the trust model.

The results suggest that the expectations of involving and being involved are somewhat different, whereas the need for information and the wish to do what is best for the service user is of common interest to both the next of kin and the frontline workers. However, it seems that the involvement process as practiced by frontline workers could be understood as more of a request for something, rather than a wish for involvement and shared decision making. The next of kin seem to be constantly wishing to be involved, and they show appreciation when they are contacted, especially when they are informed and kept in the information loop. However, the results seem to support the perception that involvement of next of kin occurs when the frontline workers need information about the service user or next of kin performing practical tasks. The literature indicates that next of kin have different roles that vary in different situations [24, 30]. This study emphasises that next of kin can be sources of expert knowledge of the service user, perform care, and serve as representatives. The results also highlight potential unspoken expectations of the frontline workers, who place multiple roles on the next of kin as interpreters, purchasers, facilitators, and important information mediators between the services in primary healthcare or hospitals. To do so, it seems that a reasonable involvement process with the next of kin should include a clarification of expectations regarding which roles are considered appropriate, possible, and desirable to hold for the next of kin in the collaboration regarding the services around the service user.

Although the guidelines regulate and emphasise the involvement of next of kin, the process of doing so seems to be unclear in our findings. Frontline workers execute the regulations to the best of their abilities, balancing factors such as large workloads, time pressure, and professional and personal evaluation of the service users’ everyday situations [26, 31, 32]. The trust model focuses on trust and professional autonomy in tailoring services, decision making, and the involvement of the next of kin [8, 20]. However, our study provides a nuanced picture of this autonomy, being bound by regulations and result orientation [26, 31, 32]. The findings further show that frontline workers are driven by unspoken expectations regarding collaborating with next of kin. In the relationship with the next of kin, mutual implicit expectations exist, which raises the question of what the expectations derive from and why it is seemingly difficult to bring into the collaboration. This can be related to assumptions and differential interpretations of the guidelines and what the collaboration and tailoring of the services should be [32]. This also suggests an ongoing negotiation of the social, cultural, governmental, and professional contexts within which the frontline workers operate [32, 33], typically facing a demand for their services that exceeds their capacity to sufficiently meet. However, studies show that next of kin also struggle to maintain autonomy and dignity on behalf of the service users while negotiating healthcare services about which they may have little knowledge [30, 34]. These findings are consistent with our results and could be understood as a driver of unspoken expectations for both the next of kin and the frontline workers. The next of kin have an expectation of being informed and involved to be able to make what is believed to be the right decisions on behalf of the service user. The frontline workers expect the next of kin to be updated and informed of the possibilities, the assessment, and criteria for the service allocations and deliveries. The next of kin seem to be striving for a position as an equal partner in the cooperation between them, to be recognised as an expert on the service user, and to have the opportunity to be a cocreator of interventions where it is perceived as advantageous.

This poses further questions regarding who holds the power to decide when and why involvement should happen. Due to different circumstances, continuous decisions need to be made with and around the service users, and the power to make the final decisions rests with the frontline workers [31]. Ultimately, they are responsible of assessing the needs and knowing the possibilities and limitations, law regulations, professional guidelines, ethics, priorities, experience, and the economic frames. These regulations make it difficult to meet the ideal involvement, and it seems that frontline workers resort to patterns of practice that are feasible within the available resources [31, 32]. The tension between these regulations and available resources is an important cause of imbalance, creating a feeling of powerlessness among the frontline workers that can cause them to practice in ways they are opposed to, such as failing to be responsive to the next of kin’s needs and wishes [7, 32, 35, 36]. Thus, instead of empowering the next of kin in the decision making of the service tailoring as intended by the trust model, the working conditions of frontline workers might constitute why the next of kin feel that their power is restricted.

The trust models’ intention enhances the importance of the involvement of service users and their next of kin when tailoring the services. It appears that from the frontline workers’ perspective, involvement serves a requesting purpose with self-interest, and contact with next of kin holds lesser value. Instead, it seems to fulfil frontline workers’ specific need for something. Hence, this leads to an understanding that the frontline workers’ and next of kin’ respective expectations of involving and being involved are somewhat different and that this in turn determines the degree of shared decision. The challenges associated with service delivery, such as time available, resource allocation, and economic constraints, also needs to be acknowledged and understood in the context of service delivery. [26]. The next of kin addresses these limitations and show understanding of how these limitations can hinder the involvement process.

The consideration of an efficient and financially sustainable healthcare service, or the requirement for professionally acceptable services, may affect the involvement of next of kin [24]. Further, frontline workers must respond to the main task, which is to deliver and tailor sufficient and necessary healthcare to the service user [32]. Frontline workers are continuously negotiating to cope with these challenges, and this study implies that they also need to be responsive to potentially conflicting wishes from the next of kin. Not being able to react to their expectations, unspoken as they seem to be, creates dissatisfaction among the next of kin, which might lead to an involvement process characterised by demands and, to some degree, struggling relations.

### Strengths and limitations

The authors varied professional backgrounds, clinical experiences, and research expertise - as occupational therapists and nurses with experience in home-based healthcare, primary care, hospital settings, and academia – have been a strength in this study. This diversity of perspectives enabled to delve deeper into a more latent level of analysis, exploring implicit meanings. It further allowed to connect the themes, existing knowledge, practice, research field, and the broader context [29]. The data material is large and rich being a combination of four interviews and 32 observations and includes quotations from a wide range of participants such as frontline workers and next of kin However, the study could have been strengthened if we had managed to recruit a larger number of next of kin. It would also be a strength if the observations were conducted after the interview enabling us to have a greater focus on the next of kin involvement.

To strengthen the trustworthiness of the study, different user involvement processes have been applied. As this study is based on a needs-led research process, the scope is developed by the research field, and the home-based healthcare services designed some of the questions asked the next of kin to ensure more relevance for the results of this study. However, no next of kin was invited to respond to the interview guide. This could have given a broader nuance to the questions asked. Efforts have been made to create a nuanced portrayal of experiences and practices by combining interviews with next of kin and observations in different meetings and settings within home-based healthcare services. Despite striving for a larger number of next-of-kin interviews and recruiting over a period of eight months, only four were successfully recruited. There were few differences in the demographics of the participants. They had different time lengths regarding collaboration with the services, but other than that, they were quite similar. This gives limited knowledge about the diversity and complexity of next of kin’s experience with involvement in home-based healthcare services.

The trust model is also a limited study setting since few municipalities in Norway have implemented the model. Several European cities have implemented a version of the model but not as interdisciplinary as it has been done in Norway. Despite this, the findings might still offer useful implications relevant to the delivery of home-based healthcare despite the organisational model since involvement and collaboration with next of kin will always be important issues to address.

## Conclusion

The trust model is an organisational model of home-based healthcare services that seeks to strengthen next of kin involvement. This study shows the complexity of the involvement processes and how unspoken expectations appear in the complex and ongoing construction of collaboration between the next of kin and the frontline workers. It points to varying motivations and desires that guide the involvement process. Specifically, a request for information or assistance with a task appears to serve as a catalyst for frontline workers, prompting the involvement process. By contrast, a desire for collaboration and shared decision making appears to drive involvement from the next of kin’s perspective. This study indicates that despite an interdisciplinary organisational model aiming for shared decision making as the trust model, the involvement of next of kin continues to be a challenge for home-based healthcare services.

Several questions seem necessary to ask in the discussion regarding the involvement of next of kin. How can the frontline workers be able to meet their expectations of involvement, and what is considered reasonable to expect? Additionally, it is crucial to evaluate the resources available to address the needs of next of kin and to design a sustainable involvement process that is beneficial while delivering flexible and individually tailored services. Our results emphasise the importance of transparent communication to elucidate implicit expectations. We suggest further studies that explore sustainable involvement strategies and recommend a co-production approach that considers the experiences and preferences of both frontline workers and next of kin.

### Electronic supplementary material

Below is the link to the electronic supplementary material.

Supplementary Material 1

## Data Availability

The dataset derives from observations and interviews, all in Norwegian, and are not publicly available because of the terms of the data collection approval but parts of the data can be made available from the corresponding author upon reasonable request.
