# Supplementary material for: Unspoken expectations and situational participation: a qualitative study exploring the instantiation of next of kin involvement within the trust model
Source: BMC Health Serv Res. 2024 Jul 30;24:866. doi: 10.1186/s12913-024-11338-9 (PMC11290214; doi:10.1186/s12913-024-11338-9)
Supplement: Supplementary file 1 — Supplementary Material 1 [file 12913_2024_11338_MOESM1_ESM.docx]

| **Interview guide** | **Individual Interviews with next of kin of service-users of home-based healthcare services** |
| --- | --- |
| Aim | Creating insight into how next of kin experience the intention of involvement, and the flexibility and adaptability of home-based healthcare services, within the context of the trust model. |
| Who | Next of kin |
| Time for interview | 45-60 minutes |
| Place | Digitalt via zoom eller over telefon, evnt. fysisk om det er ønskelig. |
| **Spørsmål** | |
| Sociodemography, Education, and Work Experience | 1. Kjønn: 2. Alder: 3. Arbeid utenfor hjemmet: 4. Hvis ja, hvor stor stillingsprosent? 5. Forhold til ditt familiemedlem (Ektefelle/Samboer/sønn/ datter/ svigerbarn/ barnebarn/søsken/ annet) |
| 1. | **How long has your close one been receiving home services, and how many hours of help are received per week?**  - Which services are you in contact with?  - Have you noticed any changes in the quality of the service in recent years? If yes, in what way?  - Time?  - Fewer helpers coming in? |
| 2. | **Involvement: In what way do you feel that you are able to involve or take part in the assessment and evaluation of the need for, and the design and delivery of the services?**  **-** What comes to mind when you hear the question "What is important to you?" Do you have experience with this question being asked?  - In what way would you say that your family is able to participate in setting the goals for the services? Do you know what the goals are?  - How are you involved in deciding what measures your mother/father/spouse receives? Are adjustments made along the way?  - Are you familiar with the content of the decision about health care? |
| 3. | **How have you experienced the contact with the service/municipality; from the time you reported a need for assistance, to the implementation of services and up until now?**  - Information  - Participation  - Has the contact changed over time? More/less dialog?  - Communication and how does it take place?  - Could you say a bit about how you experience the dialog with the service?  - How do you experience it if you provide feedback about the services given?  - Which channels do you use when you need to contact the service? Phone, email, SMS, ‘innbyggertorget’, ‘HelseNorge’ or similar? |
| 4. | **How do you perceive the competence of those who assist your close one on a daily basis? How do you experience their collaboration regarding the services in order to adapt them to needs?**  - Do you feel there are multiple professional groups involved?  - Is additional expertise brought in as needed?  - Contact with the general practitioner? |
| 5. | **Having a smaller team with fewer staff for the user to relate to is supposed to be a measure in the trust model to achieve this individual tailoring of services to those who receive them. How do you experience this?**  - What does it mean to you that the services should be flexible and individually adapted?  - Do you feel that there are familiar personnel who come by, and that you have a specific person who is responsible for the service? How do you experience this?  - Who do you contact when you need to receive/give information and how do you experience this contact?  - Do you feel that you have insight into what is happening with and around the user? Do you receive enough information about the help that is being provided? |
| 6. | **When it comes to being able to help shape the services your close one needs, and considering the collaboration with the municipality; what would you say is the optimal situation for you as a next of kin?** How should we have set up our service for you to experience this? Do you have any examples? Either positive or possibly some you wish were done differently? |
